# Performance of machine learning algorithms in spectroscopic ellipsometry data analysis of ZnTiO_3_ nanocomposite

**DOI:** 10.1038/s41598-023-50620-4

**Published:** 2024-01-18

**Authors:** Ali Barkhordari, Hamid Reza Mashayekhi, Pari Amiri, Süleyman Özçelik, Ferhat Hanife, Yashar Azizian-Kalandaragh

**Affiliations:** 1 Faculty of Physics, Shahid Bahonar University of Kerman, Kerman, Iran; 2 Department of Engineering Sciences, University of Mohaghegh Ardabili, Namin, Iran; 3 Department of Engineering Sciences, Faculty of Advanced Technologies, Sabalan University of Advanced Technologies (SUAT), Namin, Iran; 4 Department of Physics, University of Mohaghegh Ardabili, P.O. Box 179, Ardabil, Iran; 5 Department of Photonics, Faculty of Applied Sciences, Gazi University, 06500 Ankara, Turkey; 6 Photonics Application and Research Center, Gazi University, 06500 Ankara, Turkey

**Keywords:** Materials science, Optics and photonics, Optical physics

## Abstract

In this research, the optical properties of the PVP: ZnTiO_3_ nanocomposite are studied using the spectroscopic ellipsometry technique. The preparation procedure of the ZnTiO_3_ nanocomposite is explained in detail. The absorbance/transmittance, surface morphology, structural information, chemical identification, and surface topography of the ZnTiO_3_ nanocomposite is studied using UV–Vis spectroscopy, field-emission scanning electron microscopy, Raman spectroscopy, Fourier transform infra-red, and atomic force microscopy, respectively. The ellipsometry method is used to obtain the spectra of the real and imaginary parts of the dielectric function and refractive index in the photon energy range of 0.59–4.59 eV. Moreover, using two machine learning algorithms, namely artificial neural network and support vector regression methods, the ellipsometric parameters ψ and Δ are analyzed and compared with non-linear regression. The error and accuracy of each three methods, as well as the time required for their execution, are calculated to compare their suitability in the ellipsometric data analysis. Also, the absorption coefficient was used to determine the band gap energy of the ZnTiO_3_ nanocomposite, which is found to be 3.83 eV. The second-energy derivative of the dielectric function is utilized to identify six critical point energies of the prepared sample. Finally, the spectral-dependent optical loss function and optical conductivity of the ZnTiO_3_ nanocomposite are investigated.

## Introduction

Recently, metal oxides have been extensively utilized as semiconductor materials in various fields such as non-linear optics, optoelectronics^1^, electronics, power storage devices^2^, catalysts^3^, etc. So far, the photocatalytic response of some of the metal oxide materials including SnO_2_, TiO_2_, and ZnO has been studied and it has been found that most of them are only active in the UV range because of their wide band gap^4–6^. Moreover, several perovskite compounds including BaTiO_3_, CaTiO_3_, SrTiO_3_, and ZnTiO_3_ have been applied in sensors, memory instruments, and as catalyst electrodes in fuel cells, owing to their ferroelectric and piezoelectric features. Because these compounds are non-toxic and chemically stable materials that reveal a good photo reactivity in the visible region, they can be appropriate alternatives for TiO_2_^7,8^.

ZnTiO_3_ is a composite of ZnO and TiO_2_ substances, both of which are wideband semiconductors with unique features and many applications^9–11^. ZnO has multiple advantages that have been extensively discussed in the literature, including special qualities and numerous applications in transparent electronics, spin electronics, ultraviolet (UV) light emitters, chemical sensors, and piezoelectric instruments^12–15^. Compared to other materials frequently employed as semiconductors for blue-green light-emitting devices, such as ZnSe (22 meV) and GaN (25 meV), ZnO has an impressive exciton binding energy (60 meV)^16^. On the other hand, TiO_2_ is a semiconductor that is non-toxic, stable in aqueous solutions, and reasonably priced. The significant photocatalytic feature of the TiO_2_ substance originates from the wide band gap and long lifetime of photogenerated electron–hole pairs^17^. However, TiO_2_ does not use as much of the solar spectrum as other photocatalytic materials, and it also exhibits a relatively high rate of electron–hole recombination. These problems can be resolved and the activity of TiO_2_ photocatalysts improved by ZnO doping^18^.

Therefore, despite some disadvantages associated with the ZnO and TiO_2_ materials separately, these issues can be addressed through their combination, known as ZnTiO_3_. It belongs to the hybrid ZnO-TiO_2_ system, improving the optical efficiency of TiO_2_ substance by reducing the charge recombination, altering the energy band gap, and therefore shifting the optical response from UV to visible regions^19,20^. Nonlinear optical and luminous materials^21^, Catalysts^22^, microwave dielectrics^23^, nanofibers^24^, phosphors^25^, gas sensors^26^, white pigments^27^, and antibacterial stone coatings^28^ are just a few of the numerous uses for ZnTiO_3_. There are different methods to produce the ZnTiO_3_-based substances in the form of fibers, mesoporous materials, ceramics, films, and powders such as solid-state reaction^29^, chemical bath deposition^30^, molten-salt technique^27^, radio-frequency sputtering^21^, sol–gel procedure^23^, and evaporation-induced self-assembly technique^31^.

As well known, polymer molecules are long chemical chains. Thin films produced through polymer synthesis have attracted attention due to their numerous advantages, such as high stability, affordability, easy processing, and extensibility^32^. Because of these benefits, polymers are gaining popularity in the electronic and optoelectronic industries^33^. Nevertheless, the utilization of polymers may result in a decrease in electrical conductivity. Doping these polymers with metal/metal oxide nanostructures increases their electrical conductivity. Polyvinylpyrrolidone (PVP) is a non-toxic and non-ionic macropolymer widely used in the synthesis of nanocomposites^34^. Depending on the synthesis procedures and particular material systems, PVP can be used as a growth modifier, surface stabilizer, declining agent, and nanostructure dispersion. Also, it has an appropriate electronic conductivity compared to other polymers^35^.

It should be mentioned that the main parameters in the optical characterization of substances are refractive index, extinction coefficient, dielectric functions, band gap energy, and absorption coefficient, known as optical functions. The optical functions measurement is very important due to not only their importance in investigating macroscopic and microscopic materials' characteristics but also in designing and producing optical devices. One of the widely used optical techniques to measure the substances' optical functions is ellipsometry. Ellipsometry, as a non-destructive method, preserves the specimen, but accurately reproducing specimen various materials' optical coefficients is difficult in reality. Mathematically, the determination of optical functions is an inverse problem and there is no possibility for analytical solutions to the inverse ellipsometric problems in general, especially in the problems related to the samples with more than one film on a substrate. So, commonly the regression data fitting methods are utilized to get values of optical parameters that most correspond to the observed data. It is evident that these methods are based on trial and error and without a good initial estimate, achieving convergence in the fitting process is not feasible. Therefore, the intervention of an expert is required to provide an appropriate initial guess. On the other hand, sometimes it is necessary to combine multiple models for an accurate description of a sample, in which the appropriate initial estimate becomes more challenging due to the increased number of correlated fitting parameters. To overcome these problems, using new methods that there is no require for expert intervention is essential. One of the newest applicable methods is Machine Learning (ML).

ML, as a branch of artificial intelligence, was introduced by A. Samuel in 1959^36^. It enables computers to automatically process and classify existing datasets, and data analysis without the need for programming, solely based on certain algorithms. Based on the available information, the machine utilizes a specific algorithm for mathematical modeling of the given complex problem to analyze data. Artificial Neural Network (ANN), as one of the ML algorithms, processes information like the way a human brain processes. ANN includes a large number of neurons that are linked to each other, known as nodes, in various processing units similar to the brain neurons. The ANN layers are known as input, output, and hidden layers. After initial information about the considered topic to study is received in the input layer, analyzing and processing of data is done in one or more hidden layers, and then the results as ANN response are transferred to the output layer. One of the primary attributes of an ANN is its ability to extract significant information from intricate data sets that may contain noise and the ANN algorithm yields output promptly after being trained once in advance. ANN can be applied for both classification and regression purposes and has great applications in economics, forensics, pattern detection, etc. One of the other applicable ML algorithms is the Support Vector Regression (SVR) algorithm. The SVR, which was proposed by Vapnik et al.^37^, is a supervised machine-learning technique that is commonly employed for classification and regression tasks. The desirable purpose of the SVR is to construct an optimal decision boundary, referred to as a hyperplane, that in a high dimensional space separates data into various categories. In the SVR, the choice of support vectors as extreme vector points helps to form a convenient hyperplane. The distance between the hyperplane and each class's nearest data points, known as the margin, is maximized and at the same time, the classification errors are minimized. Thereby the accurate classification of data points into their befitting categories gets easier. The SVR algorithm is widely utilized in various domains such as face recognition, text categorization, image classification, self-driving cars, chatbots, etc.

Based on our knowledge and investigations, there is no spectroscopic ellipsometry data of the ZnTiO_3_ nanocomposite. In this work, the nanostructure and nanocomposite of ZnTiO_3_ are initially prepared. UV–Vis spectroscopy, field emission scanning electron microscopy (FESEM), Raman spectroscopy, Attenuated Total Reflectance (ATR), Fourier-transform infrared spectroscopy (FTIR), and atomic force microscopy (AFM) are employed to study the absorbance/transmittance, the surface morphology, structural information, chemical identification, and surface topography of the ZnTiO_3_ nanocomposite, respectively. Next, the optical constants of the ZnTiO_3_ nanocomposite are determined by the spectroscopic ellipsometry measurement in the photon energy range of 0.59–4.59 eV. To investigate and prove the efficiency of the ML approach for analyzing the ellipsometry parameters (ψ and Δ), the ANN and SVR algorithms are used in the photon energy range of 0.59–4.59 eV. Furthermore, the accuracy and time-needing of the ML methods are calculated to evaluate them and to compare the two methods' efficiency with each other as well as with the non-linear (NL) fitting method. Then, the spectral-dependent optical constants of the ZnTiO_3_ nanocomposite are calculated by the ellipsometry measurement data. The energies of the band gap and inter-band transition of the sample are also determined. Eventually, the optical loss function and optical conductivity of the ZnTiO_3_ nanocomposite will be plotted and discussed.

## Experimental details

### Synthesis of ZnTiO_3_ nanostructures and preparation of its composite

**Figure 1:**
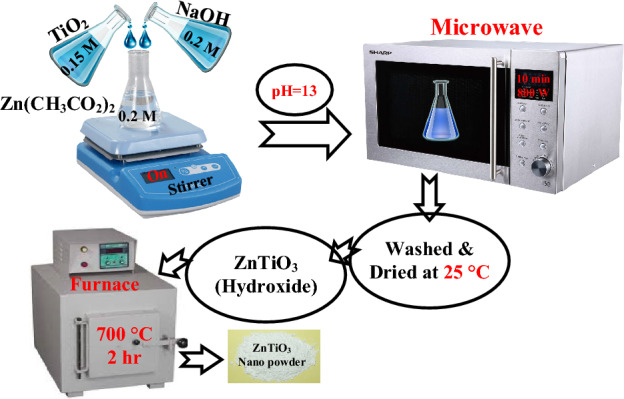
Schematic of the preparation procedure of ZnTiO_3_ nano-powder.

The precursors of TiCl_4_ (≥ 99%) and Zn(CH_3_COO)_2_.2H_2_O (≥ 99%) have been furnished by ROYALEX and Merck Companies, respectively. The details of procurement procedures of TiO_2_ nanostructures have been introduced in Ref.^38^. To provide TiO_2_ nanostructures, 20^cc^ of NaOH (0.2 M) was dropwise added to 22^cc^ of TiCl_4_ in the liquid phase on a magnetic stirrer, and after evaluating the pH of the mixture, it was irradiated by microwaves at 800W for 10 min. The distilled water was employed to wash the resulting white mixture before drying at 25 °C. For synthesizing the ZnTiO_3_ nano-powders, three separate beakers of Zn(CH_3_CO_2_)_2_ (0.2 M), NaOH (0.2 M), and TiO_2_ (0.15 M) solutions were initially made ready. Next, the solutions of TiO_2_ and NaOH were dropwise added to Zn(CH_3_CO_2_)_2_ solution at a temperature of 25 °C under ultrasonic irradiation. The product was placed in a microwave device and exposed to 800 W microwave radiation for 10 min. Then, the result was dried at the 25 °C temperature after rinsing during centrifugation. Eventually, the resulting nano-powder was annealed at the 700 °C temperature for 2 h. The preparation procedure of the ZnTiO_3_ nanostructures is schematically shown in Fig. 1.

It should be noted that a p-type Si wafer with a 300 µm thickness was employed as a substrate in this research. To prepare PVP: ZnTiO_3_ interfacial polymer layer, 10 mg of ZnTiO_3_ nanostructures was dispersed by ultrasonic technique after providing a 5% solution of PVP with solvating 5 g of PVP nano-powders in 95^ cc^ water. Then, a thin layer of PVP: ZnTiO_3_ with a thickness of 100 nm was deposited on the Si wafer using a spin coater system.

### UV-Vis spectroscopy

**Figure 2:**
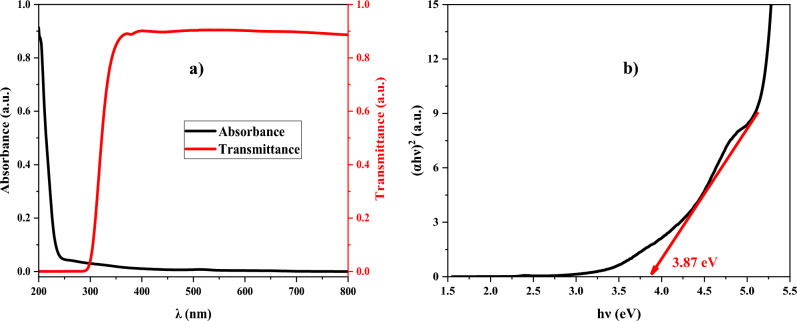
UV–Vis spectroscopy of PVP: ZnTiO_3_ nanocomposite to study the (**a**) absorbance and transmittance, (**b**) profile of (αhυ)^2^ versus hυ.

The UV–Vis spectroscopy was performed by Shimadzu UV-1800 in the wavelength range of 200–800 nm to determine the absorbance and transmittance of the ZnTiO_3_ nanocomposite (see Fig. 2a). As can be seen, the absorbance of the ZnTiO_3_ nanocomposite is decreased by raising the wavelength while the transmittance increases. In addition, Tauc’s equation is used to determine the optical band gap of the ZnTiO_3_ nanocomposite which is equal to 3.87 eV (see Fig. 2b)^38^.

### SEM Image

**Figure 3:**
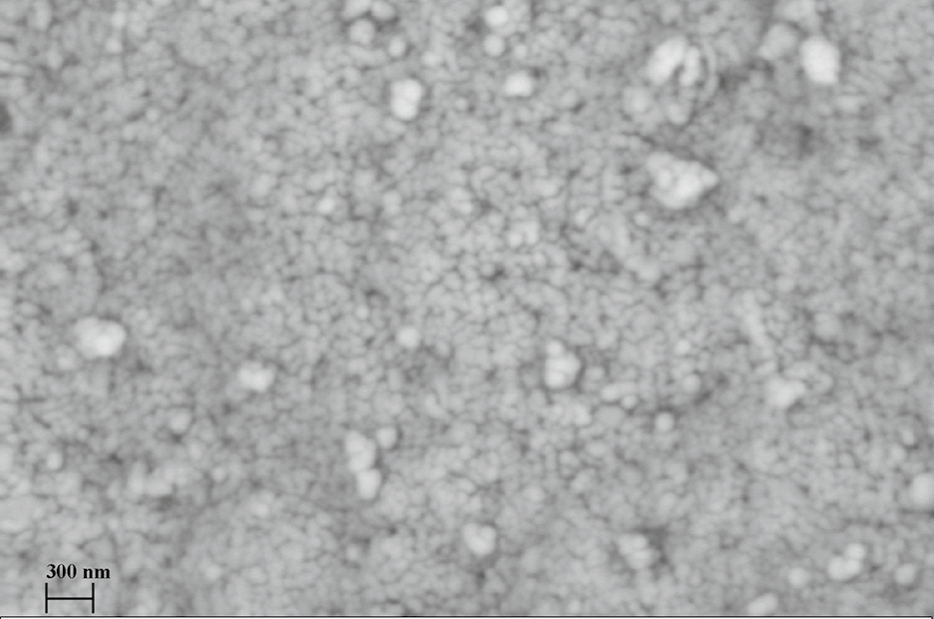
SEM image of the ZnTiO_3_ nanocomposite.

The scanning electron microscope (SEM) (model LEO 1430 VP) at 15 kV accelerator voltage was utilized to record the SEM images to survey the morphology of the ZnTiO_3_ nanocomposite. The surface-morphology schema of the prepared ZnTiO_3_ nanocomposite is depicted in Fig. 3. Because of the difficulty of observing the sizes of actual particles, the structure of polydispersity nanoparticles appears in various configurations with an average size of ˂50 nm. Furthermore, all nanoparticles are clustered to form micron-sized clusters.

### Raman spectroscopy

**Figure 4:**
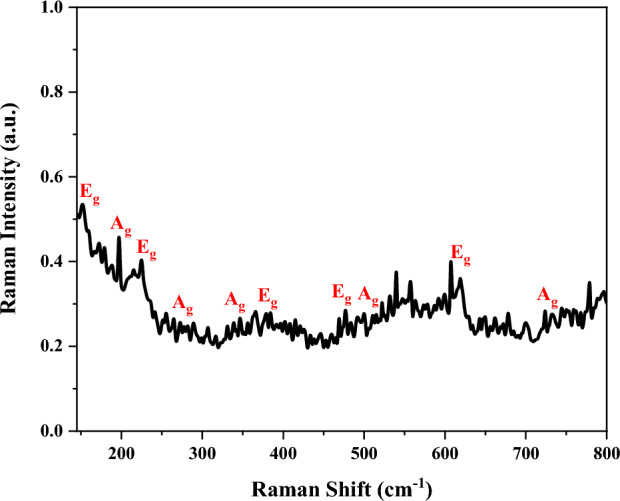
Raman spectrum of PVP: ZnTiO_3_ nanocomposite.

A potent analytical method established on the inelastic scattering of light is Raman spectroscopy, enabling unmatched structural information on the atomic scale. A high-resolution Raman spectrometer system (innoRam™) was used in this research to record the Raman spectrum of the ZnTiO_3_ nanocomposite. Based on the prediction of group theory (point group C3i), 10 Raman active modes are assigned to the ZnTiO_3_, i.e., 5A_g_ + 5E_g_^39^. Figure 4 shows the Raman spectrum of ZnTiO_3_ nanocomposite in the Raman shift of 140–800 cm^−1^. The Raman vibrations of the ZnTiO_3_ molecule were observed at the Raman shift of 152, 190, 225, 265, 346, 384, 477, 494, 619, and 724 cm^−1^ corresponded to the symmetrical phonon modes of E_g_, A_g_, E_g_, A_g_, A_g_, E_g_, E_g_, A_g_, E_g_, and A_g_, respectively. These peaks confirm the presence of ZnTiO_3_ nanostructure in the prepared PVP: ZnTiO_3_ nanocomposite. However, the observation of noise in the Raman spectrum is due to the composite of the thin layer. It is necessary to mention that low-frequency vibration modes only appeared for the particles or crystallites at nanometric size^40^. So, four peaks at 152, 190, 225, and 265 cm^−1^ corresponding to the Zn-Ti–O vibrational modes indicate the nanometric scale of the particles.

### Attenuated total reflectance (ATR) & Fourier transform infra-red (FTIR) spectroscopy

**Figure 5:**
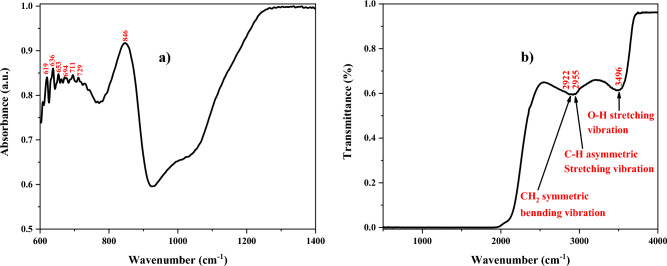
(**a**) ATR and (**b**) FTIR spectrum of PVP: ZnTiO_3_ nanocomposite.

ATR and FTIR spectroscopy methods are commonly applied for investigating the chemical identification of a desired sample. The ATR and FTIR spectra have been measured by BRUKER (vertex 80) device in this work. Figure 5 represents the ATR and FTIR spectra of PVP: ZnTiO_3_ nanocomposite with the assignment of the primary ATR and FTIR bands. Since the distinctive bands of metal oxides (< 750 cm^−1^) correspond to the bonds between metal and oxygen, the absorption band at the wavenumber range of 600–1000 cm^−1^ is attributed to the Ti–O, O–Ti–O, Zn–O, O–Zn–O, and Zn–Ti–O linked to ZnTiO_3_ species (see Fig. 5a). In more detail, the Ti–O bond vibrations are observed at the low wavenumber region from 400 to 650 cm^−1^^41^. The Zn–Ti–O vibration modes were detected at 735 cm^−1^. Moreover, the Ti–O octahedral absorption band appears at the wavenumber of 560 and 590 cm^−1^. The bands at the wavenumber of 550–650 cm^−1^ are assigned to TiO_6_ vibration modes^42^.

It is obvious that the bands at higher wavenumber regions are related to the CH_2_ symmetric bending vibration, C–H asymmetric stretching vibration, and O–H stretching vibration corresponding to 2922, 2955, and 3496 cm^−1^, respectively. It should be mentioned that the PVP is a macropolymer with CH_2_, C=O, and C–N functional groups, consisting of a very hydrophilic part and large hydrophobic groups. Since water and several non-aqueous liquids are supreme solvents for PVP, water has been used to solve the PVP polymer nano-powder in this work and hence, the O–H group appears in the FTIR spectrum (see Fig. 5b)^35^.

### Atomic force microscopy (AFM) image

The spectroscopic ellipsometry (SE) method is based on measuring the polarization of light after reflecting from a surface. It is generally accepted that optical elements do not depolarize light. However, there are certain circumstances where this assumption may not hold and depolarization can occur. For instance, when the light illuminates an area of the sample surface where the film thickness is non-uniform, it can lead to quasi-depolarization. Moreover, some of the light that reaches the polarization state detector may not have a distinguishable polarization state or cross-polarization may occur in systems that are theoretically isotropic if the sample is highly rough. On the other hand, atomic force microscopy (AFM) is an appropriate method to determine the surface roughness.

**Figure 6:**
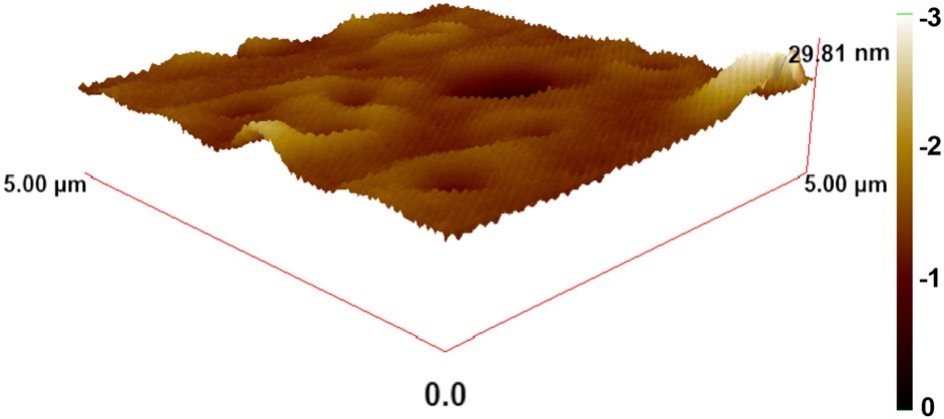
AFM image of the ZnTiO_3_ nanocomposite.

AFM, a form of scanning probe microscopy (SPM), has exhibited a resolution with an order of a nanometer compared to the optical diffraction limit. AFM uses the feeling of touching the surface with a mechanical probe to get the data. Piezoelectric components allow for small, exact movements under (electrical) control, enabling perfect scanning. It is possible to create a high-resolution image of the topography of a sample surface using the probe's response to the forces the sample imposes on it. In this work, an atomic force microscope (model WITec alpha300 A) has been employed to record the 3-dimensional shape of the surface of the PVP: ZnTiO_3_ thin film. The topography of the ZnTiO_3_ nanocomposite recorded by the AFM is represented in Fig. 6. As shown, since the ZnTiO_3_ nanocomposite's surface roughness is ⁓ 1.05 nm, this nanocomposite thin film is smooth in the sub-nanometer scale.

### Spectroscopic ellipsometry (SE) method spectroscopy

The Spectroscopic Ellipsometry (SE) technique is typically a non-invasive, non-destructive measurement method that uses reflected light waves to determine a sample material's optical properties. The SE method does not require absolute intensity as long as the absolute intensity is sufficient because it assesses a relative change in polarization. The SE measurement is exceedingly accurate and repeatable as a result^43^.

**Figure 7:**
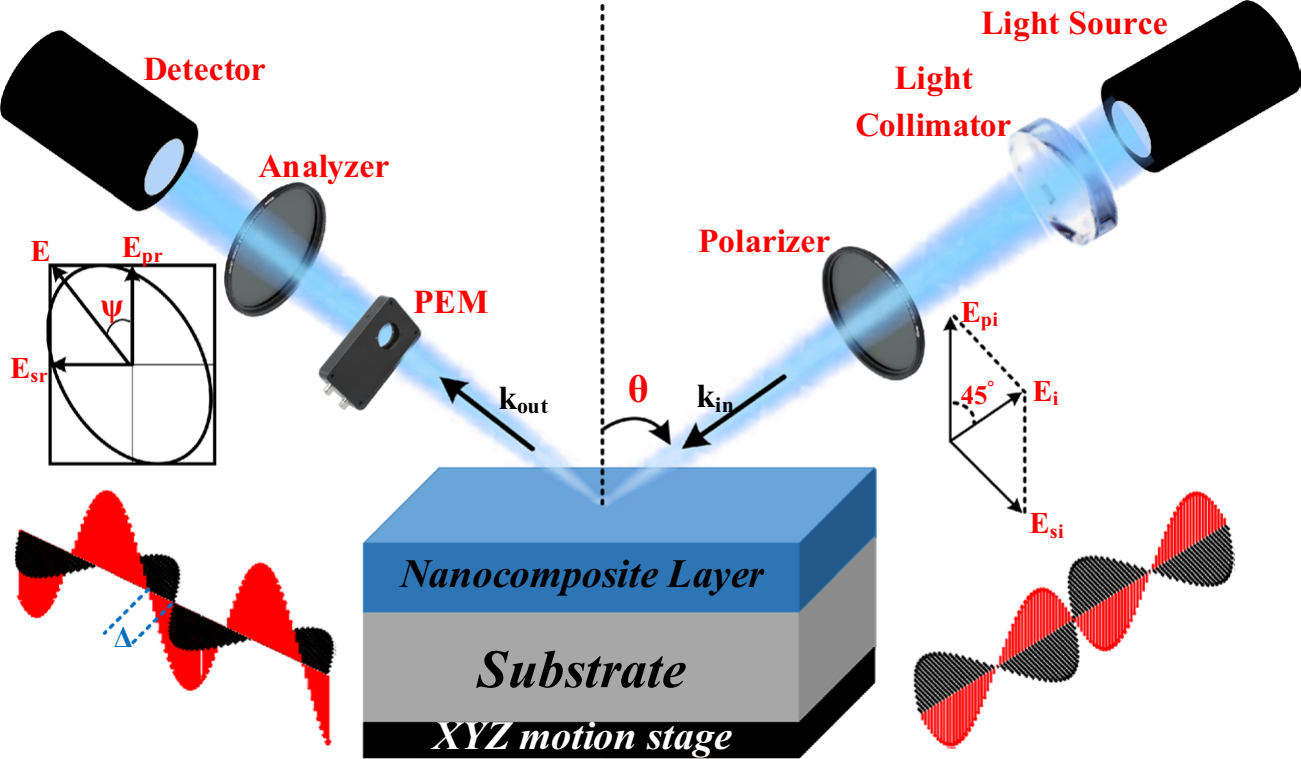
Schematic diagram of an ellipsometry setup.

Ellipsometry makes use of the fact that linearly polarized light at an oblique incidence to a surface changes its polarization state when reflected. It is elliptically polarized, giving rise to the term "ellipsometry". In addition, the incident light wave might be elliptically polarized light in some circumstances. Figure 7 depicts the general concept of ellipsometry.

Once a monochromatic plane light wave is obliquely incident on a surface, the plane of incidence is defined as a plane perpendicular to the surface, including the vector pointing in the light wave's propagation direction^44^. This vector is referred to as the wavevector **k**_**in**_. The two mutually perpendicular vectors of the light wave's electric field **E** and magnetic field **B** are perpendicular to **k**_**in**_. The only vector displayed in Fig. 7 is the **E**-vector, which is selected to represent the polarization of the light wave. The **E**-vector is divided into two parts that are perpendicular to each other and perpendicular to **k**_**in**_^44^. Figure 7 shows how the two components of **E** are parallel and perpendicular to the plane of incidence. As observed, the ellipsometer used in this work consists of seven optical elements (1) light source, (2) light collimator, (3) polarizer, (4) sample (ZnTiO_3_ nanocomposite), (5) phot-elastic modulator (PEM), (6) analyzer, and (7) detector.

The light wave that is incident to the surface is linearly polarized. The p- and s-components of the **E** field can be considered as oscillating with a specific amplitude and mutual phase whose endpoint moves in a straight line on the plane of p- and s-components^43^. The polarization of the light wave changes to an elliptical state after reflecting off the surface. As a result of the amplitude and mutual phase of the p- and s-components of **E** field changing, the endpoint of E moves in an ellipse. A detector can measure the ellipse's shape that is related to the ellipsometric parameters ψ and Δ by data processing. It should be mentioned that HORIBA (JOBIN YVON UVISEL 2) spectroscopic ellipsometer has been utilized in this work to get the ellipsometric parameters at the fixed incidence angle of 70° in room temperature. The ellipsometric parameters can be connected to the reflection coefficients of light polarized parallel and perpendicular to the plane of incidence^43^.

**Figure 8:**
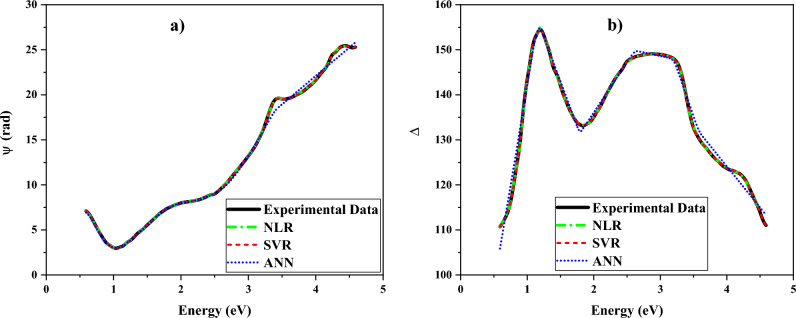
Experimentally measured ellipsometry parameters (**a**) Ψ and (**b**) Δ, compared with the spectra reconstructed by SVR, ANN, and by nonlinear regression (i.e., manual fitting).

Figure 8 illustrates the variations in the ellipsometric parameters (ψ and Δ) within the photon energy range of 0.59–4.59 eV. The experimental measurements of ψ and Δ have been analyzed not only through the NLR fitting process but also through the application of two ML algorithms, namely the ANN and SVR algorithms. As the figure shows, the fitting values are very close to the experimental ones.

To compare the obtained results more accurately, the R2 score criterion has been calculated separately for the mentioned methods. The R2 score essentially indicates the accuracy of the performed calculations, and the closer it is to the value of one, the more it signifies that the parameters considered in the algorithms were appropriate and the training was more comprehensive; Consequently, the mean deviation between the fitted values and the actual values is less. Moreover, the mean absolute error (MAE) is the other essential parameter in determining the accuracy of the analysis methods applied in this work. Table 1 introduces the value of the R2 score, MAE, and time needed to execute each of the applied algorithms for analyzing the ellipsometric parameters. The values of MAE and R2 score for the SVR algorithm are lower and higher than other algorithms in the analysis process, respectively. The time spent in ML algorithms for analyzing both ψ and Δ is lower than NLR. Moreover, the time spent in SVR is significantly lower compared to the other two algorithms.

**Table 1:** The NLR, ANN, and SVR algorithms time to model in predicting the ellipsometric angles ψ and Δ.

Algorithm	ψ (rad)			Δ		
	R2 Score	MAE	Time (s)	R2 Score	MAE	Time (s)
NLR	0.9998	0.0899	2.855	0.9962	0.7592	2.670
ANN	0.9973	0.2352	1.522	0.9852	1.1229	1.255
SVR	0.9999	0.0097	0.125	0.9999	0.0300	0.202

## Results and discussion

**Figure 9:**
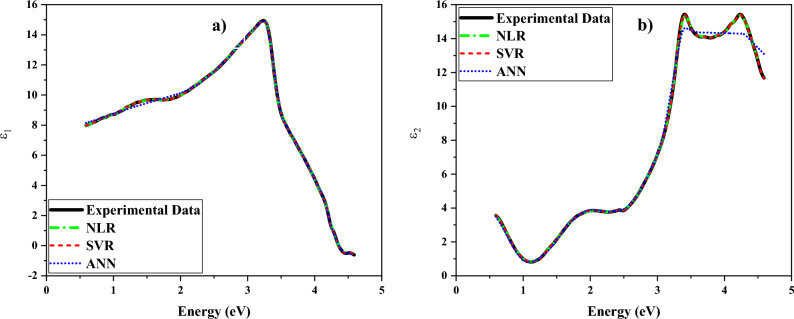
The energy-dependent (**a**) ε_1_ and (**b**) ε_2_ components of the dielectric function of ZnTiO_3_ nanocomposite determined by SVR and ANN inferences and by nonlinear regression (manual fitting regression).

The materials can be optically characterized via ellipsometric techniques. After illuminating the sample with a linearly polarized light beam, the reflected light is collected. To obtain the ellipsometric data, the amplitude ratio (ψ) of the parallel (p) and perpendicular (s) components of the reflected light, as well as the phase difference between them should be measured. The complex reflectance ratio (ρ) of the polarized light relates to the ψ and Δ ellipsometric parameters as^45^:

1 $$\rho = \frac{{r_{p} }}{{r_{s} }} = \tan \left( {\Psi } \right)\exp \left( {i{\Delta }} \right),$$

with r_p_ and r_s_ being Fresnel reflection coefficients of the reflected polarized light. The air-sample optical model to obtain the dielectric function is given by^45^;

2 $$\varepsilon = \varepsilon_{1} + i\varepsilon_{2} = \varepsilon_{0} sin^{2} \left( \varphi \right)\left[ {1 + \left( {\frac{1 - \rho }{{1 + \rho }}} \right)^{2} tan^{2} \left( \varphi \right)} \right],$$

where φ denotes the angle of incidence, ε_0_ is the dielectric constant of air, and ε_1_ and ε_2_ refer to the real and imaginary parts of dielectric constants, respectively. Figure 9 represents changes of ɛ_1_ and ɛ_2_ parts of the complex dielectric function acquired by the analysis of ellipsometric data based on the air-sample optical model. There is a maximum in the spectrum of ɛ_1_ at the photon energy of 3.24 eV. Moreover, a significant decreasing point is observed at photon energy 2 eV. Similar ɛ_2_-spectrum behavior has previously been reported in numerous ellipsometry measurements on diverse samples^46–51^. There are two peaks in the ɛ_2_-spectrum at 3.39 eV and 4.24 eV which are attributed to inter-band transitions or critical points (CPs).

Spectral-dependent refractive index (n) and extinction coefficient (k) are defined by using the spectra of ɛ_1_ and ɛ_2_ as follows^52^:

3 $$n = \left[ {\frac{{\varepsilon_{1} + \left( {\varepsilon_{1}^{2} + \varepsilon_{2}^{2} } \right)^{1/2} }}{2}} \right]^{1/2} ,$$

4 $$k = \left[ {\frac{{ - \varepsilon_{1} + \left( {\varepsilon_{1}^{2} + \varepsilon_{2}^{2} } \right)^{1/2} }}{2}} \right]^{1/2} .$$

**Figure 10:**
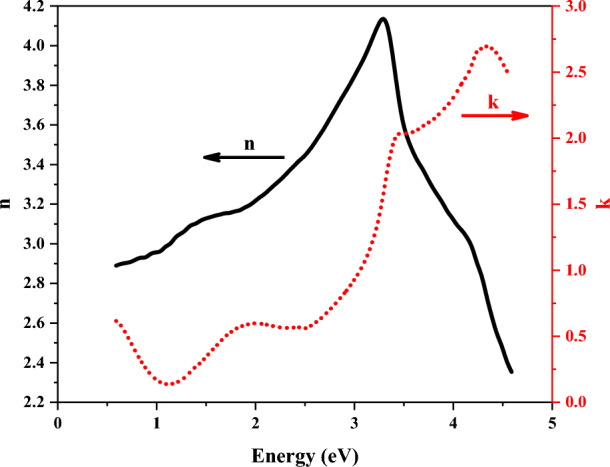
Spectral-dependent refractive index and extinction coefficient of the ZnTiO_3_ nanocomposite.

**Figure 11:**
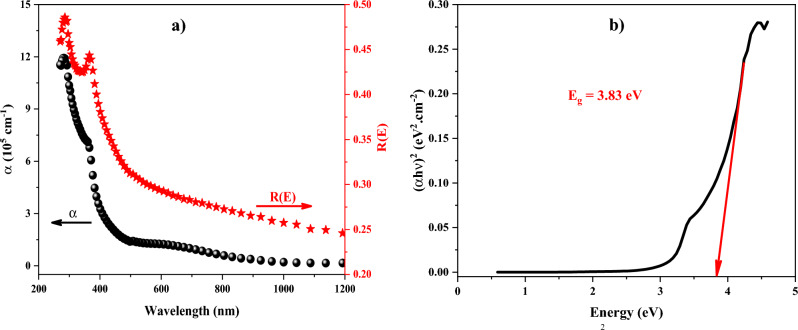
(**a**) Spectral dependencies of absorption and reflection coefficients, (**b**) (αh*v*)^2^ versus photon energy (h*v*).

Figure 10 shows the energy-dependent refractive index and extinction coefficient of the ZnTiO_3_ nanocomposite. A peak in the extinction coefficient spectrum has appeared at the same energy position of the ɛ_2_-spectrum. The band gap energy of the ZnTiO_3_ nanocomposite was calculated to be 3.83 eV which is in good agreement with the E_g_ values of 3.87 eV and 3.80 eV respectively introduced in refs.^6,53^. It can be observed that the value of the extinction coefficient is decreased at energies lower than the band gap energy. Additionally, the refractive index of the ZnTiO_3_ nanocomposite is 3.24 at the band gap energy (⁓3.87 eV), and it changes between 3.16 and 4.15 at the visible spectral region, i.e., 1.77–3.26 eV. Based on the changes of ε_2_(ω), the electron transitions from valence bands to conduction bands are what cause the peaks in the extinction and absorption coefficients (see Figs. 10, 11a).

After obtaining the refractive index and extinction coefficient of the ZnTiO_3_ nanocomposite, the absorption coefficient α(E) and orthogonal incident reflectance R(E) could be derived as^54^:

5 $$\alpha \left( E \right) = \frac{4\pi }{\lambda }k\left( E \right),$$

6 $$R\left( E \right) = \frac{{\left[ {n\left( E \right) - 1} \right]^{2} + k^{2} \left( E \right)}}{{\left[ {n\left( E \right) + 1} \right]^{2} + k^{2} \left( E \right)}}.$$

It must be noted that SE measurements on any other faces besides the crystal's usual layer-plane face are extremely challenging due to the crystal's layer organization. It is appropriate to investigate the current SE measurement spectra using the binary phase (air/sample) instance. The spectral dependencies of the absorption coefficient (α) and the reflectance coefficient (R) are introduced in Fig. 11a. The absorption spectrum has a peak and a local maximum at the wavelength of 282 nm and 360 nm, respectively. The absorption coefficient is generally declined by increasing the wavelength and it is zero in the wavelength range of 1000–1100 nm. A similar behavior could be seen in the reflectance spectrum except for zero value at no wavelength. Furthermore, the absorption coefficient and band gap energy could be related to each other as follows^55^:

7 $$\left( {\alpha h\upsilon } \right) = A\left( {h\upsilon - E_{g} } \right)^{m} ,$$

where m refers to a constant value which is 2 and 0.5 for indirect and direct transitions, respectively. Based on Eq. (7), if (αh*v*)^1/m^ is plotted as a function of h*v* at the absorption edge region, the band gap energy (E_g_) could be determined by the point where the fitted straight line intersects the energy axis. As can be seen from Fig. 11b, the indirect band gap energy for the ZnTiO_3_ nanocomposite equals 3.83 eV, where the fitted straight line intersects the energy axis. This E_g_ is in good agreement with the reported E_g_ values for ZnTiO_3_ nanocomposite^53^. These results were confirmed by the UV–Vis spectroscopy presented in Sect. 2.2.

**Figure 12:**
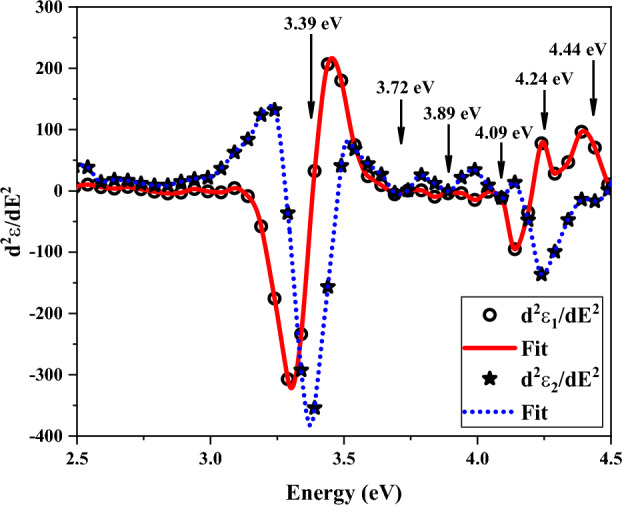
Second-derivative of the real and imaginary parts of dielectric function in terms of photon energy. Solid lines are the best fit.

According to Ref.^45^, interband transition energies (also known as critical points, or CPs) could be examined by analysis of second-energy derivatives of different dielectric function components. Theoretically, the critical point energy (E_cp_), photon energy (E), broadening parameter (Γ), amplitude (A), and phase angle (φ) relate to the second-energy derivative spectra of the components of the dielectric function as^45^:

8 $$\frac{{d^{2} \varepsilon }}{{dE^{2} }} = m\left( {m - 1} \right)Ae^{i\varphi } \left( {E - E_{cp} + i{\Gamma }} \right)^{m - 2} \;{\text{for}}\;m \ne 0,$$

9 $$\frac{{d^{2} \varepsilon }}{{dE^{2} }} = Ae^{i\varphi } \left( {E - E_{cp} + i{\Gamma }} \right)^{ - 2} \;{\text{for}}\;m = 0,$$

where m refers to the dimensions of the wave vectors involved in optical transitions. For excitonic, one, two, and three-dimensional lineshapes, the m values are 1, 1/2, 0, and + 1/2, respectively. The second-energy derivative spectra of ε_1_ (circles) and ε_2_ (stars) components are shown in Fig. 12. In the energy region, where the smoothing method has no effect on the main and first derivative spectra, a fitting process was used. As can be seen from Fig. 12, the fitting approach was used in this energy range because dielectric function components exhibit notable changes outside the 2.5–4.5 eV range.

The fitting applications had the lowest mean square deviations in the case of excitonic optical transitions (corresponding to m = -1). Six CP energies of 3.39, 3.72, 3.89, 4.09, 4.24, and 4.44 eV were obtained by fitting experimental data to theoretical definitions in the considered energy range. Near to CP energies are the observed local and absolute maximum values in the ε_2_ spectrum. These CP energies show that the sample can absorb photons with these energies.

**Figure 13:**
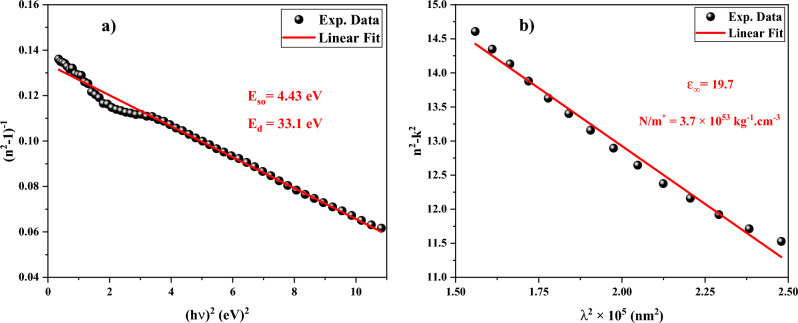
The plot of (**a**) (n^2 ^− 1)^−1^ versus (h*v*)^2^, and (**b**) (n^2 ^− k^2^) versus λ^2^.

For additional analysis, the Wemple and DiDomenico single effective model and the Spitzer-Fan model have been used to examine the refractive index and real component of the dielectric function spectra. If the photon energy is lower than the band gap energy (h*v* < E_g_), the refractive index could be expressed as follows using the Wemple and DiDomenico model^56^:

10 $$n^{2} \left( {h\upsilon } \right) = 1 + \frac{{E_{so} E_{d} }}{{E_{so}^{2} - \left( {h\upsilon } \right)^{2} }},$$

with E_d_ and E_so_ being the dispersion energy and single oscillator energy, respectively. E_so_ is described as the average band gap energy, whereas E_d_ corresponds to the intensity of the interband optical transition. To obtain the E_d_ and E_so_ values, the graph of (n^2^-1)^−1^ should be plotted as a function of (h*v*)^2^ as shown in Fig. 13a. The slope and the intercept of the linear fit are applied for calculating the value of E_d_ and E_so_ energies equal to 33.1 and 4.43 eV, respectively. Moreover, the value of the refractive index (n_0_) and dielectric constant (ε_0_) at zero frequency (*v* = 0) could be computed by using definitions of $n_{0} = \left( {1 + E_{d} /E_{so} } \right)^{ - 1}$ and $\varepsilon_{0} = n_{0}^{2}$ as 2.41 and 8.47, respectively.

The wavelength-dependent real part of the dielectric function (ɛ_1_) is given by the Spitzer-Fan model as^57^

11 $$\varepsilon_{1} = n^{2} - k^{2} = \varepsilon_{\infty } - \left( {\frac{{e^{2} }}{{\pi c^{2} }}} \right)\left( {\frac{N}{{m^{*} }}} \right)\lambda^{2} ,$$

where ε_∞_ refers to the dielectric constant at high frequencies, N denotes the carrier concentration, m* symbolizes the effective mass, c and e are the speed of light and electron charge. When n^2^-k^2^ is plotted in terms of λ^2^ (see Fig. 13b), the slope and intercept of the linear fit are used to compute the values of ε_∞_ and N/m* found as 19.7 and 3.7 × 10^53^ kg^−1^ cm^−3^, respectively.

Finally, it is useful to study the optical loss function, L(ω), and optical conductivity, σ(ω), which are defined using the dielectric function, ε(ω), as^58–60^:

12 $$L\left( \omega \right) = - Im\left( {\frac{1}{\varepsilon \left( \omega \right)}} \right) = \frac{{\varepsilon_{2} \left( \omega \right)}}{{\varepsilon_{1}^{2} \left( \omega \right) + \varepsilon_{2}^{2} \left( \omega \right)}},$$

13 $$\sigma \left( \omega \right) = \sigma_{1} \left( \omega \right) + i\sigma_{2} \left( \omega \right) = - i\frac{\omega }{4\pi }\left( {\varepsilon \left( \omega \right) - 1} \right).$$

**Figure 14:**
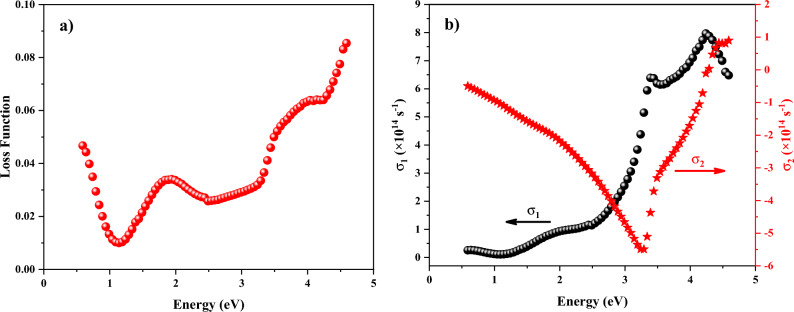
Spectral dependent (**a**) optical loss function and (**b**) optical conductivity.

Figure 14a represents the loss function of the ZnTiO_3_ nanocomposite. Generally, the energy-loss function, whose absolute maximum and matching frequencies are related to the plasma oscillations and the plasma frequency, depicts the energy loss when a fast electron passes in a material and deflects its route. As seen, the loss function of the ZnTiO_3_ nanocomposite has no absolute maximum in the considered photon energy although there is an absolute minimum at the photon energy of 1.15 eV. Figure 14b reveals the spectral dependencies of the optical conductivity of the ZnTiO_3_ nanocomposite in the considered photon energy. Optical conductivity consists of two components, i.e., σ_1_(ω) and σ_2_(ω), which are directly related to photocurrent and photoresistance^61,62^. As expected, the behavior of real and imaginary components of the optical conductivity and dielectric function are opposite. So, the ε_2_(ω) directly gives the σ_1_(ω) that indicates the absorption or energy loss in a material caused by light-induced electric current. The absorption or energy loss in the ZnTiO_3_ nanocomposite increases with the increment of the photon energy till 4.4 eV, and it reduces after that. However, a relative reduction is observed in the absorption/loss after the photon energy of 3.45 eV. The inter-band transition between unoccupied and occupied states is the main reason for the appearance of the peaks in the σ_1_(ω) spectra. On the other hand, the σ_2_(ω) is obtained by the ε_1_(ω), and it demonstrates the energy storage capacity of a material that is directly relevant to photoresistance. Therefore, the profile of the imaginary part of optical conductivity, σ_2_(ω), shows that the energy storage capacity in the ZnTiO_3_ nanocomposite is minimum at the photon energy of 3.4 eV.

## Conclusion

In this work, the spectroscopic ellipsometry method was employed to measure the optical properties of the PVP: ZnTiO_3_ nanocomposite. The details of the preparation process of the ZnTiO_3_ nanocomposite were described. UV–Vis spectroscopy, Field-Emission Scanning Electron Microscopy (FESEM), Raman spectroscopy, Attenuated Total Reflectance (ATR)-Fourier transform Infra-Red (FTIR), and Atomic Force Microscopy (AFM) were used to study the absorbance/transmittance, the surface morphology, structural information, chemical identification, and surface topography of the ZnTiO_3_ nanocomposite, respectively. The energy of the optical band gap was calculated to be 3.87 eV. It was observed that the average size of the polydispersity nanostructures was less than 50 nm. According to the group theory prediction (5E_g_ + 5A_g_), ten Raman active modes of the ZnTiO_3_ appeared in the Raman shift of 140–800 cm^−1^. The vibration modes of Ti–O, Zn-Ti–O, and TiO_6_ were detected at the low wavenumber region 400–650 cm^−1^. The functional groups of CH_2_, C-H, and O–H were also observed at the wavenumber region of 2000–3700 cm^−1^, relating to the PVP polymer and water molecules used as a solution in the preparing process of PVP: ZnTiO_3_ nanocomposite. Moreover, the roughness of the ZnTiO_3_ nanocomposite surface was ⁓ 1.05 nm, meaning the considered nanocomposite is smooth on the sub-nanometer scale.

The ellipsometric parameters (ψ and Δ) that were experimentally measured, were analyzed by the common NLR method and two algorithms of ML technique, i.e., ANN and SVR. This was done to demonstrate the efficiency of ML algorithms in ellipsometry data analysis. The obtained results show that the accuracy of data analysis using NLR, ANN, and SVR methods is very high and significant. Also, the values of the MAE and R2 score of the SVR algorithm are lower and higher than both other methods, respectively. On the other hand, the time required to analyze the ellipsometric data was calculated for all three methods. The comparison shows that the NLR method is time-consuming concerning the ANN and SVR methods. Moreover, the time needed for SVR is much less compared to the other two methods. Therefore, it can be concluded that ML algorithms can be a suitable alternative for ellipsometric data analysis, and the SVR method is preferred to the ANN method due to its low MAE, more accuracy, and less time consumption.

In addition, the spectral-dependent real and imaginary parts of the function (ε = ε_1_ + iε_2_) and refractive index (N = n + ik) were measured by the ellipsometry method at the photon energy range of 0.59–4.59 eV. The energy-dependent absorption and reflectance coefficients of the ZnTiO_3_ nanocomposite were obtained. It was found that the peaks that appeared in the absorption and extinction coefficients were due to the electron transitions from valence bands to conduction bands. Furthermore, the band gap energy of the ZnTiO_3_ nanocomposite was computed as 3.83 eV using the absorption coefficient spectrum. The second-energy derivative of the dielectric function was applied to find the six critical point energies of the prepared sample in the energy range of 2.5–4.5 eV, indicating the sample can absorb photons at these energies. Based on the Wemple and DiDomenico model, the zero-frequency refractive index (n_0_) and dielectric constant (ε_0_) of the considered sample were calculated as 2.41 and 8.47, respectively. The high-frequency dielectric constant (ε_∞_) was obtained by the Spitzer-Fan model, equal to 19.7. At last, the spectral dependencies of the optical loss function and the optical conductivity of the ZnTiO_3_ nanocomposite were studied.

## Data Availability

The data that support the findings of this study are available from the corresponding author upon reasonable request.
